# Biosynthesis and enzymology of azaindane natural products

**DOI:** 10.1038/s41429-026-00938-8

**Published:** 2026-06-29

**Authors:** Zhiyang Quan, Takayoshi Awakawa

**Affiliations:** RIKEN Center for Sustainable Resource Science, Saitama, Japan

**Keywords:** Biocatalysis, Enzyme mechanisms

## Abstract

As a representative example of biosynthetic genome mining aimed at identifying biosynthetic gene clusters whose target is unknown core enzymes for known natural products, we review the biosynthesis of altemicidin (**1**), SB-203207 (**2**), and SB-203208 (**3**). Self-resistance gene-guided genome mining led to the identification of the responsible biosynthetic gene cluster, and heterologous expression of the cluster successfully confirmed the production of compounds **1–3**. Biochemical analyses and single-gene expression studies demonstrated that the PLP-dependent enzyme SbzP is a core enzyme of this gene cluster. SbzP accepts β-NAD and SAM as substrates to generate the azaindane scaffold common to compounds **1–3**. Subsequent in vitro assays revealed the downstream tailoring reactions, involving the α-ketoglutarate-dependent dioxygenase SbzQ, GNAT-type acyltransferase SbzI, ADP-ribose transferases SbzNHO, F420-dependent reductase SbzF, SAM-dependent methyltransferase SbzE, acyl-tRNA-dependent transferase SbzA, and GNAT-type acyltransferase SbzC, which collectively modify the SbzP product to yield the final structure of compound **3**. Structural studies of SbzP and SbzI elucidated the molecular basis for substrate recognition and protein–protein interactions. This review highlights the novelty and utility of investigating biosynthetic gene clusters categorized as Group III and provides new insights into the biosynthesis of β-NAD-derived natural products.

## Introduction

Natural products are important sources of compounds for pharmaceutical development [1]. However, the discovery of novel natural products using traditional methodologies has become increasingly rare, and alternative approaches are required. Biosynthetic studies of natural products are essential not only for understanding the complex reactions leading to the final products but also for providing new insights that enable the discovery of novel compounds. Advances in next-generation sequencing technologies have made it possible to obtain vast amounts of genomic information, and genome databases currently contain extensive data on biosynthetic genes. Consequently, modern biosynthetic studies are deeply integrated with genomic information, as natural product biosynthetic enzymes are encoded within biosynthetic gene clusters (BGCs). Extracting enzyme information from genetic data greatly facilitates the understanding of enzymatic reactions [2].

**Fig. 1:**
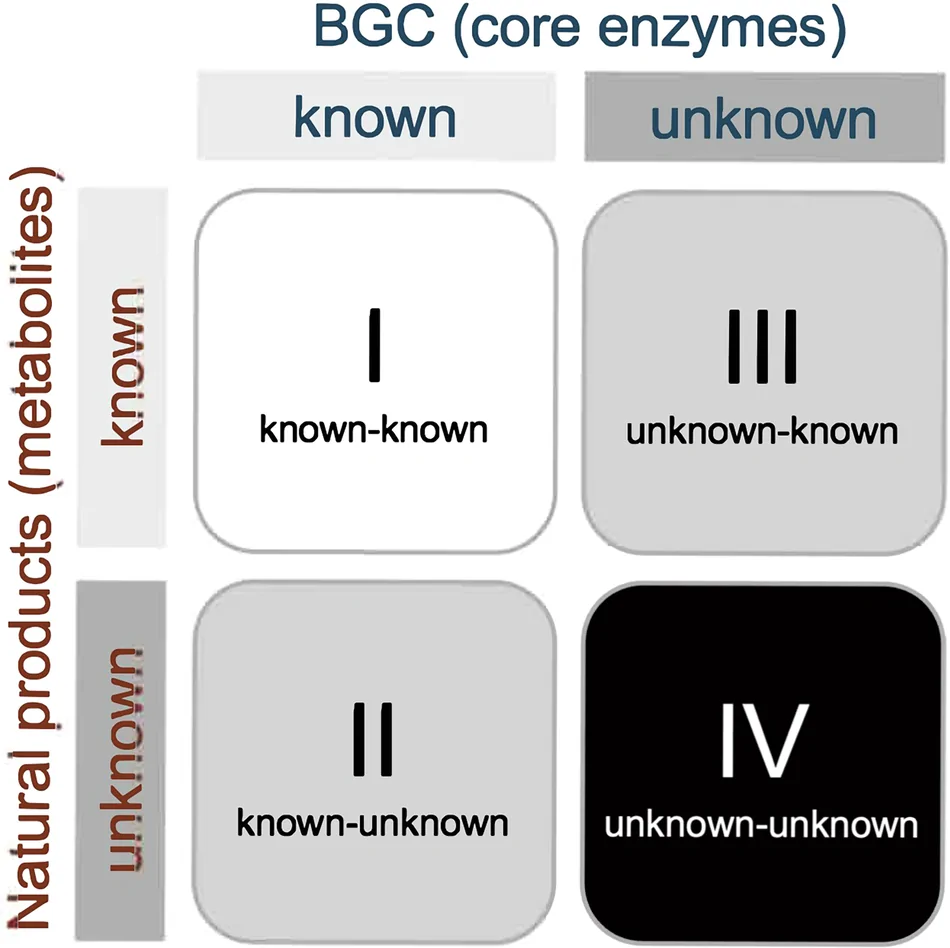
Classification of the relationship between natural products and biosynthetic gene clusters. The X-axis represents known or unknown gene clusters based on core enzyme predictions. The Y-axis represents known or unknown metabolites

Genome mining targets can be classified into four groups based on whether the core enzyme encoded by a gene cluster and/or the cluster product is known or unknown (Fig. 1) [3]. Most genome mining efforts aimed at discovering novel compounds focus on Group II targets (known core enzymes and unknown products), whereas some studies target Group III (unknown core enzymes, known products) to identify novel biosynthetic enzymes. The discovery of new core enzymes in Group III provides valuable information that can be used to identify new targets for Group II genome mining. Since gene clusters cannot be identified by focusing on an unknown core enzyme, it is often necessary to focus on tailoring enzymes that modify the core structure or, in some cases, on self-resistance proteins encoded within the gene cluster [4]. Because the products synthesized by discovered core enzymes are novel, studies of these core enzymes and their downstream enzymes offer rich opportunities to uncover new biochemical principles.

## Identification of the gene cluster for altemicidin (1), SB-203207 (2), and SB-203208 (3) biosyntheses

**Fig. 2:**
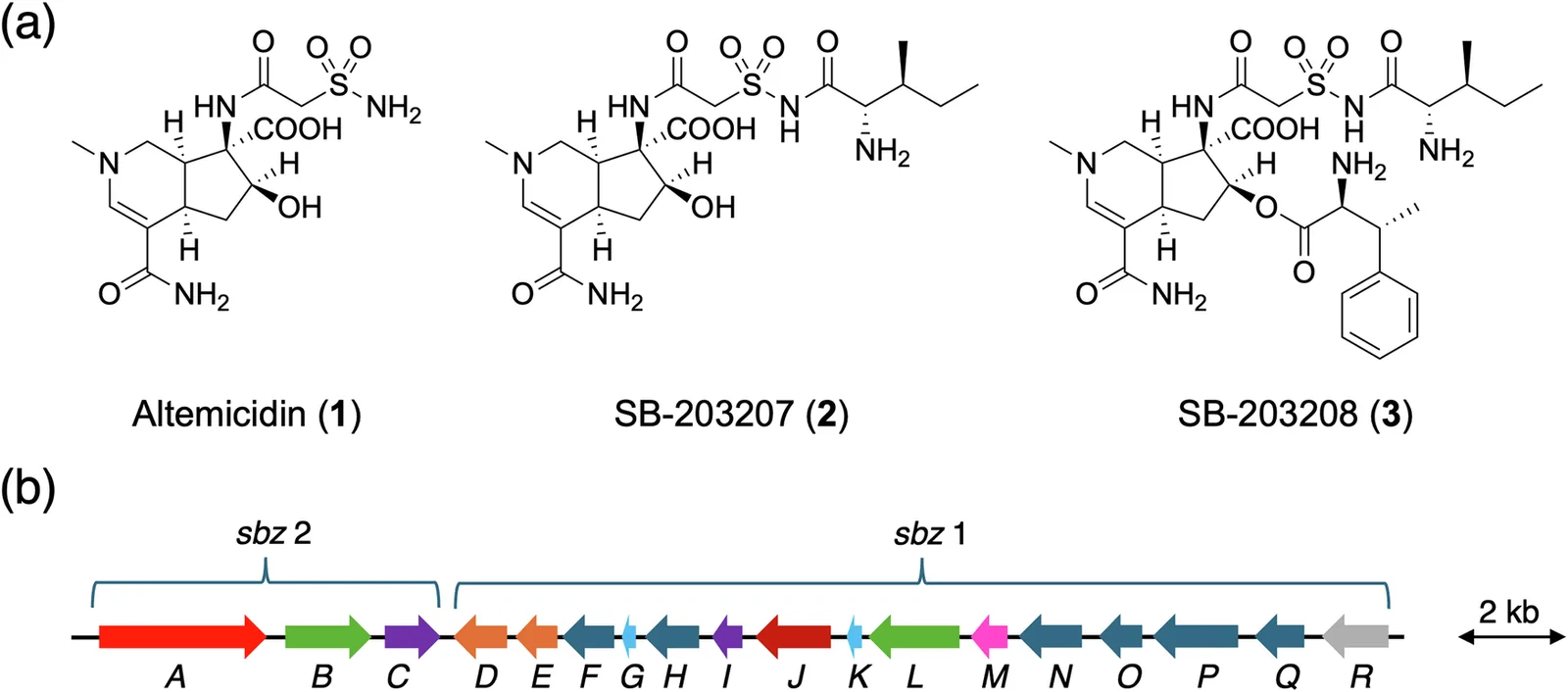
Chemical structures of azaindane natural products and their biosynthetic gene cluster. **a** Chemical structures of azaindane natural products, altemicidin, SB-203207, and SB-203208. **b** Schematic representation of the biosynthetic gene cluster for these compounds

Here, we used altemicidin (**1**), SB-203207 (**2**), and SB-203208 (**3**) as examples to identify the BGC and biosynthetic machinery corresponding to Group III targets (Fig. 2). Compounds **1**–**3** are naturally occurring alkaloids containing rare sulfonamide and azatetrahydroindane functional groups. Compound **1** was first isolated from *Streptomyces* strain SA-1758 and exhibited both acaricidal and antitumor activities [5, 6]. Compounds **2** and **3** were isolated from *Streptomyces* spp. NCIMB 40513 and are structural derivatives of **1**. Compound **2** shows potent inhibitory activity against isoleucyl-tRNA synthetase [7, 8].

Because genomic information on the core enzymes involved in the biosynthetic pathway of **1–3** could not be obtained, traditional genome mining approaches are not suitable for identifying the corresponding BGC. Therefore, putative resistance proteins encoded within the gene cluster have been considered key markers for locating the BGC responsible for **1–3** biosynthesis.

Compound **2** has been reported to be an isoleucyl-tRNA synthetase inhibitor; therefore, this enzyme is inferred to function not only as a target of **2** but also as a resistance protein. An isoleucyl-tRNA synthetase (accession number: P00956.5) encoded by the genome of *Escherichia coli* was used as a query sequence to perform a BLASTp search against the genome of *Streptomyces* sp. NCIMB 40513 to identify potential resistance genes in the producer strain. Two genes encoding Ile-tRNA synthetases were identified in *Streptomyces* sp. NCIMB 40513: *Ssp-IleRS* (27% amino acid identity) and *SbzA* (25% amino acid identity). While *Ssp-IleRS* is a housekeeping gene, *sbzA* is encoded within a 20-kb gene cluster designated *sbz* (Table 1). The *sbz* gene cluster comprises 18 genes organized into two operons, one containing three genes (*sbz-2*) and the other containing 15 genes (*sbz-1*) (Table 1) [9].

**Table 1:** The *sbz* gene cluster for SB-203208

Gene	Amino acid	(base pairs)	Proposed function	Closest protein homologue	Positive	Identity
*sbzA*	1033	(3102)	Isoleucine-tRNA synthetase	Q5YYW9 (*Nocardia farcinica* IFM 10152)	70%	57%
*sbzB*	532	(1599)	AMP-binding enzyme	Q9R9J0 (*Bacillus subtilis*)	53%	36%
*sbzC*	346	(1041)	GNAT family enzyme	Q47LA0 (*Thermobifida fusca* YX)	61%	41%
*sbzD*	334	(1005)	MppJ like methyltransferase	Q643C8 (*Streptomyces hygroscopicus*)	56%	40%
*sbzE*	260	(783)	Methyltransferase	P54458 (*Bacillus subtilis* subsp. subtilis str. 168)	48%	27%
*sbzF*	320	(963)	F420-dependent glucose-6-phosphate dehydrogenase	E8NCH3 (*Microbacterium testaceum* StLB037)	45%	30%
*sbzG*	85	(258)	Peptidyl carrier protein	Q89VT6 (*Bradyrhizobium diazoefficiens* USDA 110)	63%	39%
*sbzH*	334	(1005)	Aldose 1-epimerase	P05149 (*Acinetobacter calcoaceticus*)	53%	35%
*sbzI*	188	(567)	GNAT family enzyme	C7IZ16 (*Oryza sativa* Japonica Group)	37%	28%
*sbzJ*	464	(1395)	Gamma-aminobutyraldehyde dehydrogenase	Q6D6Y7 (*Pectobacterium atrosepticum* SCRI1043)	52%	39%
*sbzK*	93	(282)	Peptidyl carrier protein	A7ZA76 (*Bacillus velezensis* FZB42)	49%	36%
*sbzL*	559	(1680)	AMP-binding enzyme	O07610 (*Bacillus subtilis* subsp. subtilis str. 168)	43%	28%
*sbzM*	228	(687)	Cupin	A1WGK0 (*Verminephrobacter eiseniae* EF01-2)	45%	35%
*sbzN*	385	(1158)	Sugar isomerase	Q8TZ14 (*Methanopyrus kandleri* AV19)	41%	27%
*sbzO*	268	(807)	BtpA	Q58515 (*Methanocaldococcus jannaschii* DSM 2661)	51%	27%
*sbzP*	523	(1572)	Aminotransferase	Q0S962 (*Rhodococcus jostii* RHA1)	56%	34%
*sbzQ*	304	(915)	Dioxygenase	Q94FY7 (*Arabidopsis thaliana*)	54%	38%
*sbzR*	409	(1230)	MFS transporter	O34546 (*Bacillus subtilis* subsp.subtilis str.168)	36%	24%

The chemical structure of **3** reveals the presence of several distinct moieties, including a unique sulfonamide, a tetrahydronicotinamide, an uncharacterized C4 amino acid residue, an L-isoleucine residue, and a β-methylphenylalanine residue. Several of these moieties are methylated, such as the tetrahydronicotinamide and β-methylphenylalanine residue. Consistent with these structural features, the proteins encoded within the *sbz* gene cluster were functionally associated with the biosynthesis and further modification of these moieties. For example, SbzK and SbzG are putative peptidyl carriers, SbzB and SbzL are similar to adenosine monophosphate-binding enzymes, and SbzC and SbzI are homologs of the general control non-repressible 5-related *N*-acetyltransferase (GNAT) family enzymes (Table 1) [10]. These enzymes are likely involved in the installation of amino acid building blocks, such as β-methylphenylalanine and l-isoleucine. SbzD and SbzE belong to the class of *S*-adenosylmethionine (SAM)-dependent methyltransferases and are proposed to catalyze the *N*-methylation of nicotinamide and the *C*-methylation of β-methylphenylalanine. Indeed, the methyltransferase MppJ involved in β-methylphenylalanine biosynthesis has been reported [11], and it shows 40% identity to SbzD encoded in the cluster. Although the functions of some enzymes encoded within this cluster remain uncharacterized, these collective observations support the conclusion that the *sbz* gene cluster represents a putative BGC for **3**.

To identify the products of the *sbz* cluster, two operons, *sbz-1* and *sbz-2*, were introduced into *Streptomyces lividans* TK21 for heterologous expression. Expression of *sbz-1* resulted in the production of **1**, whereas co-expression of *sbz-1* and *sbz-2* led to the production of **2** and **3**, indicating that the enzymes encoded in *sbz-1* are responsible for azaindane biosynthesis and those encoded in *sbz-2* are responsible for amino acyl transfer.

## Identification of the reaction catalyzed by a core enzyme, PLP-dependent transferase SbzP

Because the *sbz* gene cluster is a cryptic BGC, the available bioinformatic information on this BGC is insufficient to propose a detailed biosynthetic pathway for **3**. The authors first identified the gatekeeping enzyme of the BGC. Each gene within the *sbz* gene cluster was heterologously expressed in *S*. *lividans* TK21, and the resulting products were analyzed. Only the expression of *sbzP*, which encodes a PLP-dependent transferase, led to the production of a specific metabolite, thereby leading to its identification as nucleoside compound **4**. Accordingly, SbzP was identified as the gatekeeping enzyme encoded by the *sbz* BGC [12].

The authors conducted feeding experiments using *S*. *lividans* harboring the operon *sbz-1* to identify SbzP substrates. l-(¹³C₄,¹⁵N)Aspartic acid and (¹³C₃)glycerol were supplied to the cultures, and the resulting metabolite **1** was analyzed by ¹³C NMR and ¹H,¹⁵N-HMBC spectroscopy. Consequently, significant incorporation of isotopically enriched precursors into **1** was observed. These results clearly demonstrate that the nicotinamide moiety is derived from l-aspartic acid and glycerol, consistent with nicotinamide adenine dinucleotide (NAD) biosynthesis in *E. coli* [13], whereas the uncharacterized amino acid moiety is derived from l-aspartic acid and forms a five-membered ring via a [3 + 2] annulation reaction catalyzed by SbzP.

**Fig. 3:**
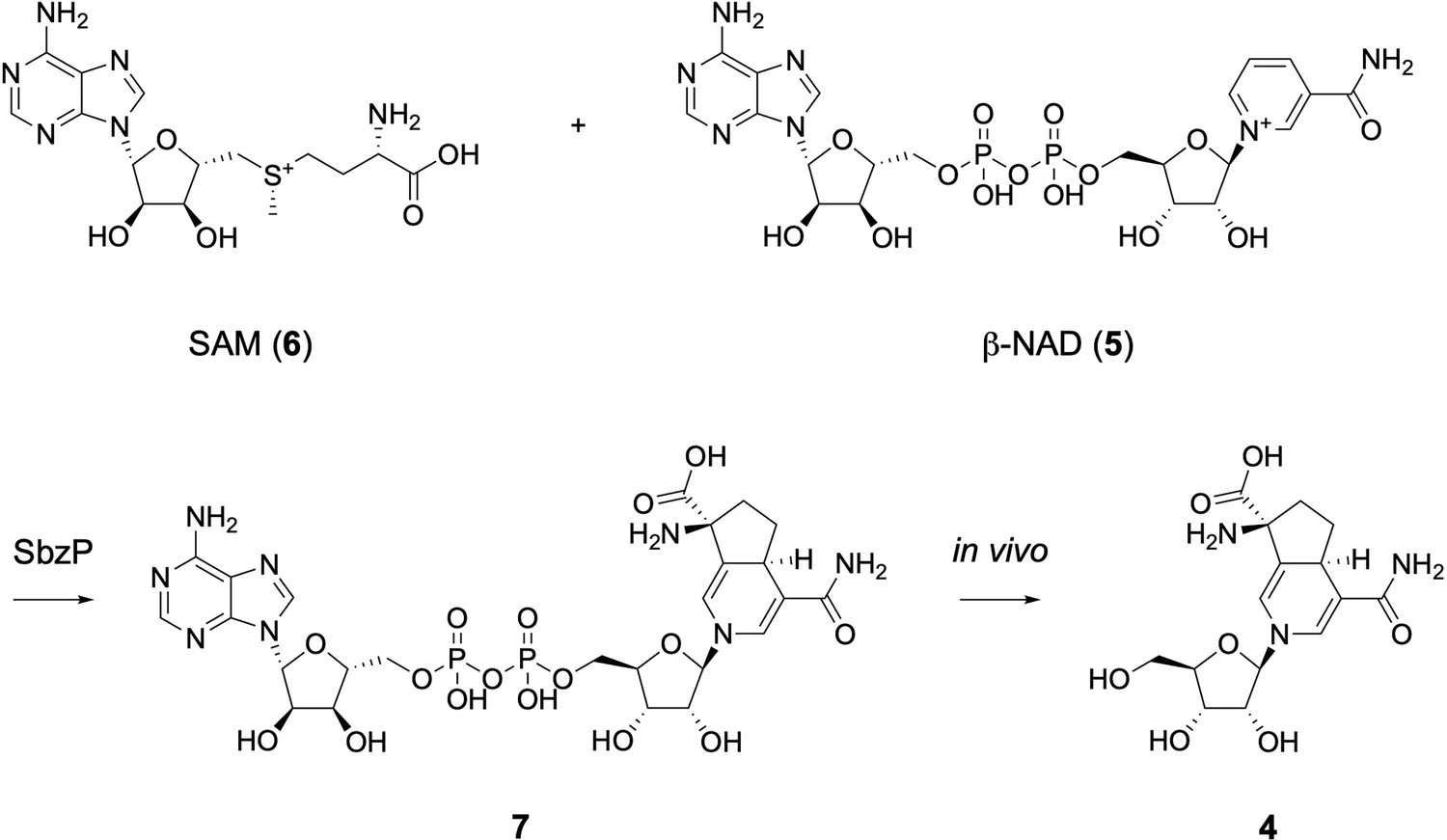
The reaction catalyzed by SbzP to produce **7** from β-NAD and SAM

Based on the results of the isotope feeding experiments, β-nicotinamide mononucleotide (β-NMN), which is metabolically derived from aspartic acid and glyceraldehyde 3-phosphate, was initially hypothesized to be a substrate of SbzP. The authors conducted enzymatic reactions in which β-NMN with nine candidate α-amino acid substrates was individually incubated with SbzP. However, no significant substrate consumption was observed, indicating that β-NMN is not a substrate for SbzP. As the electrophilic *N*-ribosyl pyridinium moiety provides sufficient reactivity toward nucleophilic addition, β-NAD and β-NAD phosphate, which are dinucleotide analogs of β-NMN, were subsequently hypothesized to be substrates for SbzP. Enzymatic assays were conducted using β-NAD or β-NAD phosphate in combination with the nine candidate α-amino acids. Among these combinations, specific substrate consumption was detected only in the reactions containing β-NAD and SAM. These results indicated that the true substrates of SbzP are β-NAD and SAM. The product of the SbzP-catalyzed reaction was isolated and structurally elucidated as dinucleotide **7** (Fig. 3).

## Identification of downstream reactions from SbzP

**Fig. 4:**
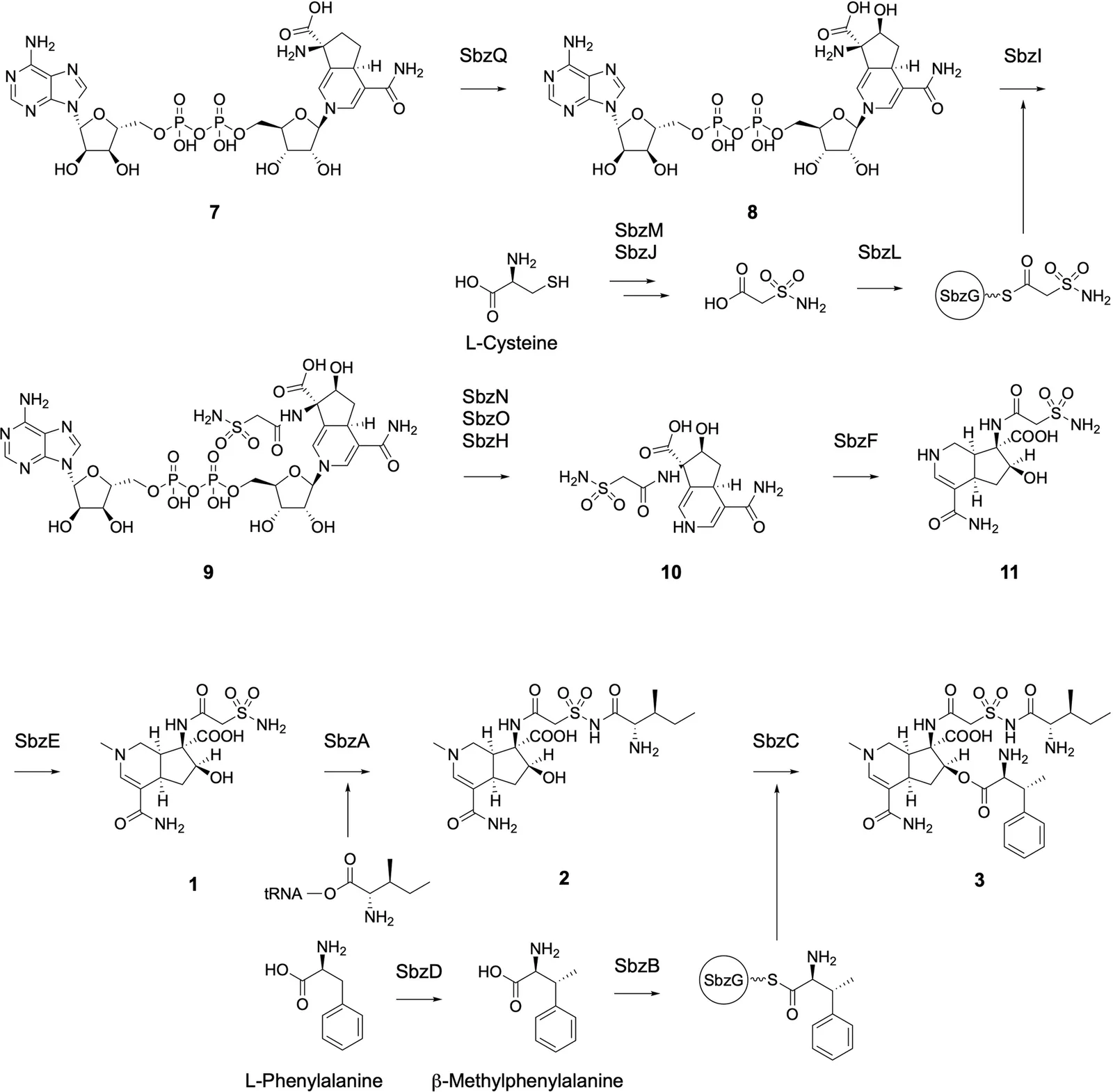
Biosynthetic pathway of SB-203208 (**3**)

The authors performed gene deletion in the heterologous expression system and in vitro approaches to elucidate the complete biosynthetic pathway of **1**. The biosynthesis of **1** is initiated by an SbzP-mediated reaction, in which β-NAD (**5**) serves as one substrate, and SAM (**6**) serves as the other. In this reaction, SAM first reacts with PLP to form an intermediate, which subsequently reacts with β-NAD, finally leading to the formation of a five-membered ring of **7**. Compound **7** is then oxidized at C2 by SbzQ, an α-ketoglutarate-dependent monooxygenase [14–16], to generate 8. Subsequently, a sulfonamide moiety is introduced into **8** to generate **9**. SbzI (a GNAT family transferase [10]), SbzM (a cupin dioxygenase [9, 17–19]), SbzJ (an NAD-dependent oxygenase [20]), and SbzG (or SbzK, both of which are peptidyl carrier proteins) are involved in this transformation. The sulfonamide moiety is biosynthesized from l-cysteine using SbzM and SbzJ. Next, SbzN (a sugar isomerase [21]), SbzO (a BptA-like protein [22]), and SbzH (a galactose mutarotase [23]) catalyze the subsequent steps, leading to the formation of **10** (Fig. 4). During these reactions, the adenosine pyrophosphate group is removed, and the ribose moiety adjacent to nicotinamide is cleaved, leaving the nicotinamide, amino acid, and sulfonamide residues in **10**. Compound **10** is reduced using SbzF (an F₄₂₀-dependent oxidoreductase [24]) to produce **11**. A methyl group is subsequently transferred to the nitrogen atom of the six-membered nicotinamide ring using SbzE (an *N*-methyltransferase), yielding **1**. An l-isoleucine residue is then transferred to the sulfonamide by SbzA (an isoleucyl-tRNA synthetase [9, 25]) to generate **2**. Finally, a β-methylphenylalanine residue is installed onto the hydroxyl functional group at C2 by SbzC (a GNAT family transferase) together with SbzG (or SbzK), producing **3**. β-Methylphenylalanine is derived from l-phenylalanine and undergoes *C*-methylation, catalyzed by SbzD (an MppJ-like methyltransferase [11]) (Fig. 4).

## Structure-function analysis of SbzP

Within this complex biosynthetic pathway, the authors focused on the catalytic mechanism of SbzP, a unique PLP-dependent enzyme that mediates the intriguing [3 + 2] annulation between the nicotinamide moiety and a quinonoid derived from SAM [26–28]. During these investigations, the authors found that SbzP exhibited poor stability. Thus, PsePQ, SbzP-SbzQ fusion protein homolog from *Psuedomonas*, was selected as the target enzyme instead of SbzP owing to its high stability. PsePQ is a chimeric protein consisting of an SbzP-like domain (52% amino acid identity with SbzP) at the C terminus fused to an α-ketoglutarate-dependent oxygenase domain (a homologue of SbzQ) at the N terminus. Molecular dynamics simulations and density functional theory calculations were performed to investigate the reaction mechanism.

**Fig. 5:**
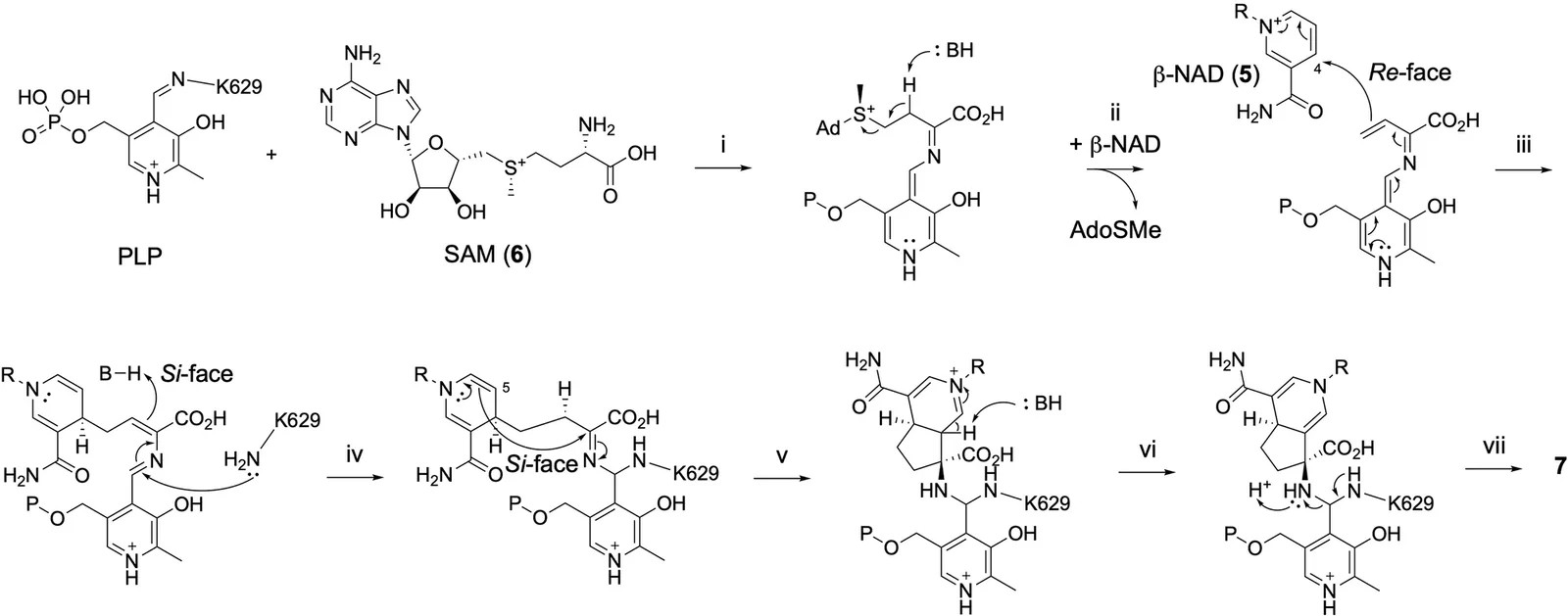
Reaction mechanism of SbzP/PseP. R = ADP-ribose, Ad Adenine, K629 = Lys629, B-H unidentified residue for proton donor

The proposed reaction mechanism is illustrated in Fig. 5. PLP in PsePQ initially formed an internal aldimine with K629. Upon substrate binding, SAM enters the active-site pocket of PsePQ and undergoes transaldimination with the PLP–K629 internal aldimine, resulting in an external aldimine. Subsequent deprotonation of Cα in SAM generates a quinonoid intermediate (Fig. 5i). A basic amino acid residue within PsePQ abstracts a proton from Cβ of the external quinonoid, triggering C–S bond cleavage and forming AdoSMe (Fig. 5ii). After AdoSMe is released from the active-site pocket, β-NAD enters the pocket and reacts with the α,β-unsaturated quinonoid. The Cγ of the olefinic moiety of the quinonoid attacks C4 of the nicotinamide ring of β-NAD from the *Re* face, forming a C–C bond between the β,γ-unsaturated quinonoid and nicotinamide (Fig. 5iii). Subsequently, the amino moiety of K629 attacks the aldimine, triggering a cascade of electron-pair rearrangements, resulting in reprotonation of Cβ from the *Si* face (Fig. 5iv). The C5 of the nicotinamide ring then attacks Cα from the *Si* face to form a five-membered ring (Fig. 5v). The basic amino acid residue of PsePQ then abstracts a proton from C5 of the nicotinamide ring, generating a deprotonated intermediate (Fig. 5vi). Finally, transaldimination restores the PLP–K629 internal aldimine to its initial state, and compound **7** is released as a product derived from SAM and β-NAD.

**Fig. 6:**
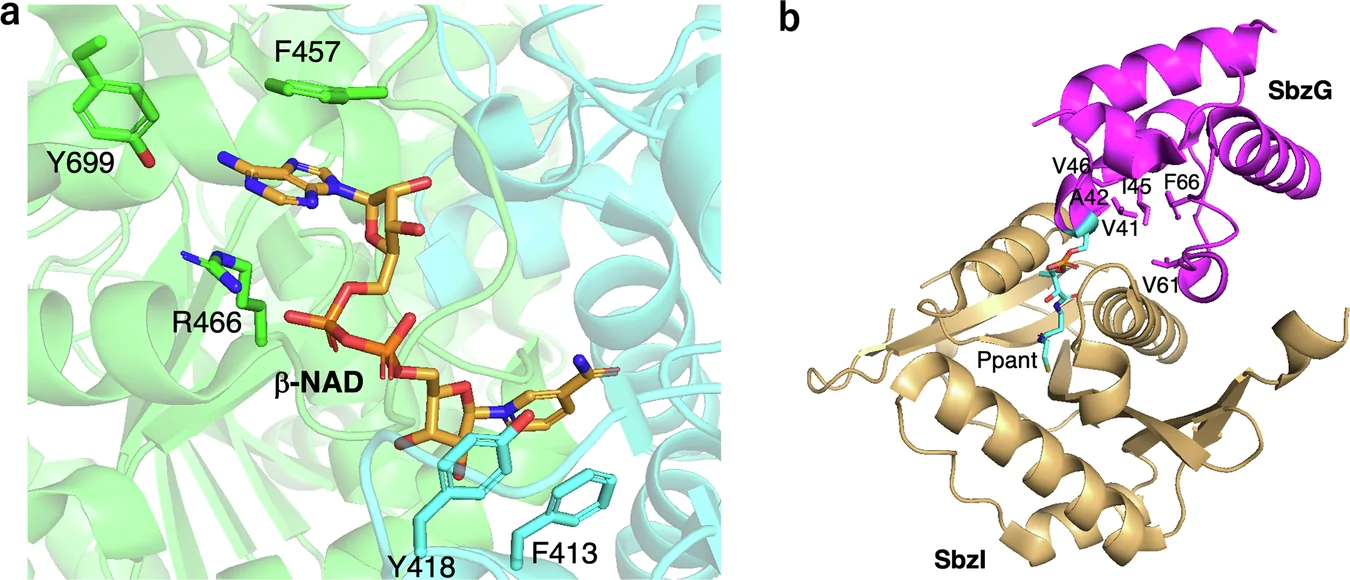
Structure analysis of **1**-**3** biosynthetic enzymes. **a** β-NAD binding mode in PseP. **b** The complex structure of SbzI and SbzG

The authors employed cryo-EM to determine the structure of this protein. β-NAD, β-NADH, SAM, *S*-adenosyl-l-homocysteine, aminovinylglycine, vinylglycine, and sinefungin were used as ligands for cryo-EM analysis. Consequently, the ligand-free structure of PsePQ was resolved at 2.83 Å, and the structure of PsePQ in complex with β-NAD was determined at 2.85 Å [28]. The other ligands yielded no interpretable structures. Mutagenesis studies were also conducted on PsePQ. Combined analysis of the cryo-EM structures and mutagenesis data indicated that the amino acid residues Y699, F457, R466, Y418, and F413 play important roles in β-NAD binding (Fig. 6a), whereas Y699, F457, and R466 are critical for SAM binding. Furthermore, K629 was identified as essential for PLP transamination and catalysis.

## Structure-function analysis of SbzI

SbzI is a GNAT superfamily enzyme that catalyzes the transfer of a 2-sulfamoylacetyl (2-SA) group to the amine moiety of compound **8**. While most GNAT superfamily enzymes utilize acyl-coenzyme A (acyl-CoA) substrates, SbzI uniquely recognizes a carrier-protein (CP)-tethered 2-SA substrate [29]. The authors investigated SbzI through a crystallization approach to reveal the protein–protein interaction between SbzI and SbzG, the CP involved in altemicidin biosynthesis. In addition, the authors engineered SbzI to expand its substrate scope and generate novel azaindane dinucleotide derivatives.

To elucidate the protein–protein interaction mechanism between SbzI and SbzG, the authors employed a linker-based protein connection, in which the C terminus of SbzG was connected to the N terminus of SbzI via a flexible Gly–Gly–Gly–Gly–Ser linker to form the fusion protein SbzGI. Crystallization of both SbzI and SbzGI was performed, and a comparison between apo-SbzI and holo-SbzGI revealed the interaction mechanism between SbzI and SbzG. The two proteins bind to each other through a combination of hydrophobic interactions, salt bridges, and hydrogen bonds at the protein–protein interface. Structural analysis and mutagenesis studies indicated that V41, A42, I45, V46, V61, and F66 of SbzG are involved in hydrophobic interactions with SbzI and are critical for protein recognition by SbzG (Fig. 6b). The crystal structures further revealed that 4′-phosphopantetheine (Ppant) is attached to S38 of SbzG and that the Ppant arm inserts into the activesite pocket of SbzI (Fig. 6b).

**Fig. 7:**
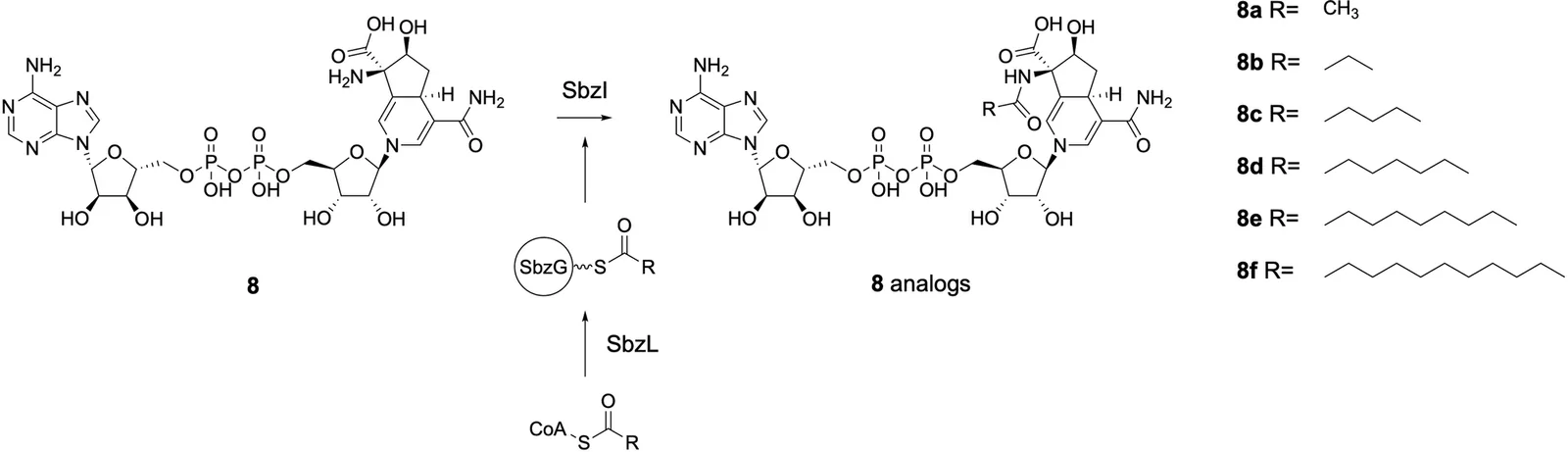
Substrate specificity of SbzI

As the 2-SA-binding space is sufficiently large for substrates with carbon chain lengths of four carbon atoms or longer, the authors evaluated a series of fatty acyl-CoAs, including C2, C4, C6, C8, C10, C12, and C18 acyl-CoAs, as substrates. Malonyl-CoA, methylmalonyl-CoA, succinyl-CoA, aminoacyl-CoA, prolyl-CoA, and alanyl-CoA were also tested as potential substrates. Consequently, C2-, C4-, and C6-CoA were accepted by SbzGI (Fig. 7 Compounds **8a**, **8b**, and **8c**). Furthermore, the aminoacyl-CPs alanyl-SbzG and prolyl-SbzG were accepted by SbzI. By contrast, long-chain acyl-CoAs, such as C8-, C10-, C12-, and C18-CoA, were not accepted by SbzGI, nor were malonyl-SbzG or methylmalonyl-SbzG accepted by SbzI.

Comparative analysis of the acyl-binding sites of SbzI and N-acyl amino acid synthase FeeM, a homologous GNAT enzyme capable of accepting C12 acyl-CoA as a substrate, indicated that the amino acid residues Leu99, Leu120, and Leu150 in SbzI formed the bottom surface of the active-site pocket and restricted the length of the acyl donor. To expand the substrate scope of SbzI, each of the three residues was substituted with l-alanine. Among the resulting variants, the L150A mutant accepted C8- and C10-acyl substrates and produced the novel compounds **8d** and **8e**. Furthermore, double mutants L99A/L150A, L120A/L150A, and S137A/L150A were constructed, enabling the acceptance of C12 fatty acyl–SbzG substrates and leading to the generation of an additional novel compound **8f**.

During this step of the biosynthetic pathway (from **8** to **9**), 2-sulfamoylacetic acid is transferred to **8** by SbzI, with the assistance of the carrier protein SbzG, to generate **9**, as described previously. The biosynthesis of 2-SA involves SbzM and SbzJ. This pathway is initiated by l-cysteine, with SbzM catalyzing multiple oxidation steps at the sulfur atom, followed by SbzJ, which catalyzes the oxidation of the aldehyde intermediate to the corresponding carboxylic acid [9, 20].

## Summary and outlook

The BGC responsible for the production of azaindane natural products **1–3** was identified using a self-resistance gene-guided approach. Since SbzA has been shown to exhibit typical isoleucine tRNA synthetase activity [9], its primary role is likely that of an isoleucine synthetase; however, it can still be considered to function as a resistance protein. Since BGCs do not always encode self-resistance proteins, this strategy is not universally applicable for identifying clusters whose core biosynthetic genes cannot be readily recognized. In such cases, complementary approaches, including metabolite-based screening and gene expression regulation strategies, are necessary. Compound **2** was identified as an inhibitor of isoleucyl-tRNA synthetase, suggesting that analogs of 2 discovered through genome mining based on SbzA homologs may represent a new class of acyl-tRNA synthetase inhibitors. Homologs of SbzP are widely distributed across more than 200 bacterial genomes and metagenomes. Most of these homologs conserve the β-NAD-binding site identified in cryo-EM structural studies, indicating that they are likely to produce products identical or highly similar to those generated by SbzP. By contrast, the sequence identities between SbzI and its homologs are relatively low, raising the possibility that azaindane compounds bearing diverse side-chain acyl groups, distinct from those of 3, remain to be discovered. In addition, the presence of auxiliary biosynthetic enzymes—such as radical SAM enzymes [30, 31], α-ketoglutarate/Fe(II)-dependent dioxygenases [14–16], and PLP-dependent enzymes [26, 27]—within the gene cluster is expected to further expand the structural diversity of the resulting metabolites. Because no reports exist on the isolation of natural products derived from β-NAD other than compounds **1-3**, the isolation of a novel class of natural products from the products of the gene cluster containing SbzP is highly promising. Given the widespread distribution of gene clusters, their products are expected to play important biological roles beneficial to survival. Another notable feature of this biosynthetic system is the formation of sulfonamide moieties catalyzed by the cupin dioxygenase SbzM. Genome mining efforts focusing on SbzM homologs may enable the discovery of previously unexplored natural sulfonamide products [18]. Although the cytotoxicity of compound **1** has been previously reported [5], its molecular targets remain unknown. The identification of these targets, together with structural optimization guided by binding models of azaindane compounds, is expected to enhance their potential as pharmaceutical agents. Furthermore, the biosynthetic insights obtained from these studies will facilitate the structural diversification of azaindane compounds via biosynthetic and semibiosynthetic approaches, thereby supporting their future development as bioactive molecules. SbzP/PseP represents the first PLP-dependent enzyme known to catalyze a nucleophilic Cγ-addition reaction [26, 27]. Expansion of the substrate scope of these enzymes would enable the creation of novel cofactor-derived compounds. Because several SAM-accepting PLP enzymes have been reported [32–36], comparative analysis between SbzP and these enzymes should provide valuable insights for protein engineering. The unnatural acylated azaindane products generated by SbzI are expected to exhibit physicochemical properties distinct from those of the SbzP products, particularly in terms of polarity, potentially leading to altered or novel bioactivities. Furthermore, engineering SbzI to transfer a sulfonamide moiety onto structural frameworks other than azaindane compounds could yield unprecedented primary sulfonamide-containing metabolites. Given the highly unusual chemical properties of the compounds produced in the biosynthesis of **1–3**, pathway engineering of this system holds significant potential for the generation of novel bioactive molecules derived from metabolic cofactors.
